# Adoptive immunotherapy against ovarian cancer

**DOI:** 10.1186/s13048-016-0236-9

**Published:** 2016-05-17

**Authors:** Gloria Mittica, Sonia Capellero, Sofia Genta, Celeste Cagnazzo, Massimo Aglietta, Dario Sangiolo, Giorgio Valabrega

**Affiliations:** Candiolo Cancer Institute-FPO- IRCCS, Candiolo, Turin, Italy; Department of Oncology, University of Torino, Turin, Italy; Division of Medical Oncology-1, Candiolo Cancer Institute- FPO- IRCCS, Strada Provinciale 142 km 3.95, Candiolo, 10060 Turin, Italy

**Keywords:** Adoptive-cell therapy, Ovarian Cancer, LAK, TILs, NK, CIK

## Abstract

The standard front-line therapy for epithelial ovarian cancer (EOC) is combination of debulking surgery and platinum-based chemotherapy. Nevertheless, the majority of patients experience disease recurrence. Although extensive efforts to find new therapeutic options, cancer cells invariably develop drug resistance and disease progression. New therapeutic strategies are needed to improve prognosis of patients with advanced EOC.

Recently, several preclinical and clinical studies investigated feasibility and activity of adoptive immunotherapy in EOC. Our aim is to highlight prospective of adoptive immunotherapy in EOC, focusing on HLA-restricted Tumor Infiltrating Lymphocytes (TILs), and MHC-independent immune effectors such as natural killer (NK), and cytokine-induced killer (CIK). Adoptive cell therapy (ACT) has shown activity in several pre-clinical models. Available preclinical and clinical data suggest that adoptive cell therapy may provide the best benefit in settings of low tumor burden, minimal residual disease, or maintenance therapy. Further studies are needed to better define the optimal clinical setting.

## Background

Epithelial ovarian cancer (EOC) is the most lethal gynecological cancer [1, 2]. Prognosis of early-stage ovarian cancers is favorable with approximately 90 % of patients surviving 5 years after diagnosis [3]. However, more than 70 % of patients are diagnosed with advanced disease (FIGO stage III-IV) [4]. Although many patients with advanced tumors initially benefit from integrated surgery and platinum based chemotherapy [5, 6], recurrence develops in nearly 90 % of cases [7, 8].

Time to progression (TTP) after front-line platinum-based therapy is one of the most important prognostic factors and it is important to define further treatments [9–11]. Patients with a TTP greater than 6 months (platinum sensitive ovarian cancers) have a more favourable prognosis and are liable to receive another platinum-based treatment. Patients with a TTP shorter than 6 months (platinum resistant ovarian cancers) have a very poor prognosis and are treated with a non-platinum-derived chemotherapy such as pegylated liposomal doxorubicin (PLD) [12, 13], topotecan [14], etoposide [15], weekly paclitaxel [16], docetaxel [17] or gemcitabine [12, 13]. The clinical benefit is marginal and similar for all these agents (around 20 %).

Regardless the type of treatment, repeated therapies favor drug-resistance through the selection of chemo-resistant clones, allowing tumor survival and progression and forcing patients to undergo several lines of chemotherapy with poor results and severe side effects.

In this context, there is a clear unmet need for alternative treatments to improve clinical outcome of advanced EOC [5].

Increasing evidence suggests that EOC is immunogenic and may be recognized and attacked by the immune system [1, 18]. Spontaneous antitumor immune response was identified in nearly half patients with advanced disease [5]. Tumor-specific lymphocytes have been identified in tumor microenvironment, in ascites and in peripheral blood were identified, capable of oligoclonal expansion, recognizing tumor antigens and displaying tumor-specific cytolytic activity in vitro [18].

**Fig. 1:**
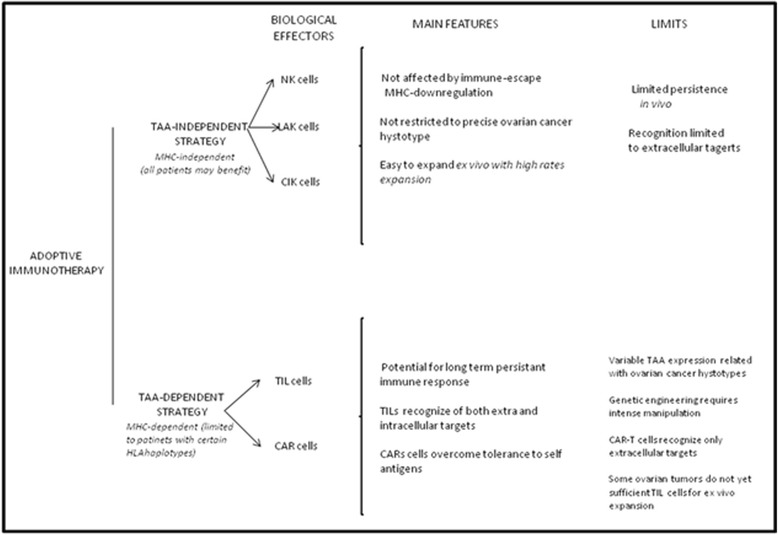
Schematic representation of the main features and limits of TAA-specific and TAA-independent adoptive immunotherapy strategies against ovarian cancer. CAR: Chimeric-antigen receptors; CIK: Cytokine-induced killer; HLA: Human leukocyte antigen; LAK: Lymphokine Activated Killer; NK: Natural killer; TAA: Tumor associated antigen; TIL: Tumor-infiltrating lymphocytes

It was reported that tumor infiltrating CD8+ effector T cells in EOC correlate with improved progression free survival (PFS) [6, 19]. On the contrary, the presence of CD4 + CD25 + FoxP3 T regulatory cells (Tregs), recruited by tumor cells, and the activation of immune evasion mechanisms (e.g., negative Immune checkpoint regulators (i.e., B7-H1 and endothelin B repressors) are associated with poor clinical outcome [4, 20–22]. Cancer immunotherapy has recently emerged as a clinically effective tool in several solid tumors [23]. Among all the possible immunotherapeutic strategies, adoptive immunotherapy is considered one of the most promising options. Adoptive immunotherapy has shown encouraging activity mainly in melanoma and soft tissue sarcomas [23] and hopes are hold for a possible extension to other histotypes such as ovarian cancer. Adoptive immunotherapy is based on the infusion of ex vivo expanded and/or activated immune effectors able to identify and destroy neoplastic cells [6, 24, 25]. Adoptive immunotherapy may be based either on HLA-restricted or unrestricted strategies [24]. The first focuses on T lymphocytes capable of recognizing tumor associated antigens (TAA) through their specific T cell receptor (TCR); the second focuses on elements of the innate immune system that that do not rely on HLA-mediated recognition of tumor targets; these effectors are natural killer (NK) cells, Lymphokine Activated Killer cells (LAKs), cytokine-induced killer (CIK) cells. Anti-tumor lymphocytes may be adoptively infused unmodified or previously engineered with TAA-specific TCRs or chimeric antigene receptors (CARs) [26]. In this review, we will focus on the preliminary clinical evidence and perspectives offered by adoptive immunotherapy in the field of EOC. To identify ongoing clinical trials with adoptive immunotherapy we operated a search on clinicaltrials.gov with “ovarian cancer” and “adoptive” as keywords. The work is dedicated to adoptive immunotherapies based on unmodified immune effectors (TILs, NK cells, LAK cells, CIK). Strategies with genetically engineered lymphocytes will not be included in the present work due to limited space and current absence of clinical evidence in EOC (Fig. 1).

## HLA restricted adoptive immunotherapy

### Tumor infiltrating Lymphocytes (TIL)

Adoptive transfer of autologous TILs has been successfully tested for the treatment of metastatic melanoma with an objective response rates (ORR) ranging up to 50 % [23, 27, 28]. Several studies confirm that T cell infiltration in epithelial tumors is associated with a better prognosis [18, 29–31]. To date, several TAAs have been described as potential targets in ovarian cancer such as [4] New York esophageal-1 (NY-ESO-1), p53, Human Epidermal Growth Factor 2 (HER2)/neu [32], survivin, folate receptor α, sperm surface protein (Sp 17), Wilms Tumor protein 1 (WT1), Mucin 1 (MUC1), melanoma associated antigen-3 (MAGE3), CA-125 and human telomerase reverse transcriptase (hTERT) [33]. In 2003, Zhang and colleagues reported a correlation between the distribution of TILs and clinical outcome in EOCs. The analysis performed in 186 frozen specimens from advanced-stage EOCs showed that the presence of CD3+ TILs was associated with a significant improvement in median PFS (22.4 vs 5.8 months) and OS (50.3 vs 18 months) [18].

Other recent studies confirmed that both CD3 and CD8+ TILs are associated with a favorable prognosis in EOC. Milne and colleagues assessed the presence of dendritic cells lymphocytes, MHC class I and II by tissue microarray analysis in high-grade serous, endometrioid, mucinous and clear cell tumors. They showed that disease-specific survival (DFS) was associated with the CD8, CD3, FoxP3, TIA-1, CD20, MHC class I and class II expression [34]. Similar results have been published by Clarke’s group on a retrospective series of 500 patients [35].

An example of functional TAA targeting in EOC is provided by in vitro cultured T lymphocytes (obtained from EOC patients or healthy donors) specific for Human Sperm protein 17 (Sp17), which is highly expressed in EOC [36]. Upon adoptive infusion in NOD/SCID mice, T lymphocytes were able to eradicate EOC tumor xenografts expressing high levels of Sp17 [37].

Emerging evidence suggests that TILs are not a monomorphic entity, but are phenotypically and functionally very different in terms of persistence, memory and antitumour activity. The understanding of this complexity is essential and might be critical in future design of clinical trials [23]. Furthermore, other important clinical issues such as systemic administration of IL-2 or lymphodepletion preparative regimens may impact of clinical trial outcome [23].

#### Clinical activity of TILs

Starting from '90s, clinical studies have investigated the efficacy of TILs in EOC. In 1991 Aoki et al. treated 17 patients with advanced or recurrent EOC [38]. Seven patients were treated with TILs after a single infusion of cyclophosphamide. In this group, TILs represented the first line therapy in 4 patients ineligible of standard chemotherapy; 3 patients had a recurrent chemo-resistant tumor. Another group of 10 patients received TILs in association with chemotherapy. Eight patients with a previously untreated tumor received cisplatin, adriamycin, 5-fluorouracil and cyclophosphamide; 2 patients with a platinum-resistant tumor received an analogue of cisplatin (254-S) as a single agent. One complete response and four partial responses were reported in the 7 patients treated with TILs infusion alone. However, duration of response was only 3–5 months. In the group of 10 patients treated with TILs in combination with chemotherapy, the authors observed 7 complete and 2 partial responses. The duration of response ranged from 13 to more than 26 months. According to the literature the response rate (RR) induced by chemotherapy is about 60 % [39, 40]. In this work TILs addition resulted in an increase of RR up to 90 %. This difference is not statistically significant, probably due to the small number of enrolled patients. Furthermore the possibility that responses in combined group might be only due to chemotherapy cannot be excluded [38].

Three years later Freedman et al. conducted a pilot study to determine the clinical effects of intra-peritoneal (i.p.) TIL expanded with recombinant IL-2 (rIL-2) plus i.p. low-dose rIL-2. Eleven patients with platinum-refractory disease were enrolled; 8 received TIL plus rIL-2 while 3 were treated with r-IL2 alone since TIL failed to expand. Grade 3 peritonitis and anemia were reported (each one occurred in 1 of 9 cycles of TIL plus rIL-2 and 1 of 38 cycles of rIL-2 alone). Even if there were no measurable responses, in 2 patients was observed ascites reduction, one patient had a tumor and CA-125 reduction and one had a surgically confirmed stable disease [41].

In 1995, Fujita et. al published a case-control trial to test the efficacy of maintenance therapy with TILs. Thirteen patients (TILs group) with no residual tumor after primary cytoreduction and platinum-based chemotherapy, received additional maintenance therapy with TILs obtained from cancer tissues. Eleven additional patients (control group) received standard treatment consisting in surgery followed by platinum-based chemotherapy. In this study, a significant difference both in 3-year OS (100 vs 67.5 % *p* < 0.01) and 3-year disease free survival (DFS) (82.1 vs 54.5 % *p* < 0.05) was observed. The 3-year DFS difference was also statistically significant in patients with macroscopic residual tumor after surgery (76.2 vs 33.3 %, *p* < 0.05). The treatment was well tolerated, with no severe complications reported [42].

#### Ongoing clinical studies

In 2010 the National Cancer Institute decided to assess if a TILs based therapy, previously given to over 200 patients with melanoma, can lead regression of digestive tract, urothelial, breast, or ovarian/endometrial tumors and to investigate the safety of his treatment. This Phase II Study Using Short-Term Cultured, autologous TILs following a Lymphocyte Depleting Regimen in Metastatic Cancers (NCT01174121) aims to enroll approximately 260 patients to determine the rate of tumor regression. The results are expected for 2019 (Table 1).

**Table 1:** Clinical Trials of adoptive immunotherapy in ovarian cancer

Sponsor	ID	Condition	Type of immunotherapy	Primary outcome	Secondary outcome	Status
University Healt Network of Toronto	NCT01883297	Recurrent, Platinum Resistant High Grade Serous Ovarian, Fallopian Tube, or Primary Peritoneal Cancer	Re-stimulated tumor-infiltrating lymphocytes (TILs)	Number occurrences and severity of side effects	Clinical response to treatment Number of patients with an immunity and no immunity to the study treatment	Recruiting
National Cancer Institute	NCT01174121	Metastatic cancer (digestive tract, urothelial, breast, ovarian/endometrial)	Re-stimulated tumor-infiltrating lymphocytes (TILs)	determine the ability of autologous TIL to mediate tumor regression	/	Recruiting
Harlev Hospital	NCT02482090	Metastatic Ovarian Cancer	Re-stimulated tumor-infiltrating lymphocytes (TILs)	Determine the safety of the administration of TIL therapy including lymphodepleting chemotherapy and Interleukin-2 for patients with metastatic Ovarian Cancer	Tumor related immunoresponses ORR OS PFS	Recruiting
Mie University	NCT02096614	Solid tumors (melanoma, head and neck cancer, ovarian cancer, esophageal cancer)	MAGE-A4 Specific TCR Gene Transferred T Lymphocytes	Confirm the toxicity profile Confirm no replication competent retrovirus observed Confirm no clonality is observed Evaluate persistence and expansion of transferred TBI-1301	/	Recruiting
Mie University	NCT02366546	Solid tumors (melanoma, head and neck cancer, ovarian cancer, synovial sarcoma, esophageal cancer)	NY-ESO-1-specific TCR gene transduced T lymphocytes	Toxicity profile confirmation Confirm no replication competent retrovirus observed Confirm no clonality is observed Evaluate persistence and expansion of transferred TBI-1301	/	Recruiting
Memorial Sloan Kettering Cancer Center	NCT00562640	Fallopian Tube, Ovarian, Primary Peritoneal Cancer	Wilms’ tumor gene (WT1) peptide sensitized autologous T cells	Safety and tolerability Mean tolerated dose of autologous WT1 peptide-specific T cells Quantitation of alterations in the concentration of peptide-specific T cells in the blood at defined intervals post infusion Effects of the adoptively transferred T cells on the growth and progression of cancer	/	Active, not recruiting
Fred Hutchinson Cancer Research Center	NCT00101257	Advanced ovarian cancer	Autologous CD4+ Antigen Specific T Cell Clones	• Safety and toxicity of autologous CD4-positive antigen-specific T cells • Determine the duration of in vivo persistence of this drug in these patients.	• Determine the antitumor effect of this drug in these patients.	Completed
Abramson Cancer Center of the University of Pennsylvania	NCT01312376	Recurrent Ovarian Fallopian Tube or Primary Peritoneal Cancer	Vaccine-Primed CD3/CD28-Costimulated Autologous T-Cells Combined With Vaccine Boost and Bevacizumab	• determine the feasibility and safety	/	Completed
Abramson Cancer Center of the University of Pennsylvania	NCT02277392	Recurrent Ovarian Carcinoma, Fallopian Tube or Primary Peritoneal Cancer	Recombinant Human Interleukin-18 (Sb-485232) Combined With Adoptive Transfer of Vaccine-Primed CD3/CD28-Costimulated Autologous T-Cells Following Lymphodepletion	• determine the feasibility and safety	/	Completed
Fred Hutchinson Cancer Research Center	NCT00003887	Breast Cancer, Chronic Myeloproliferative Disorders, Gestational Trophoblastic Tumor, Kidney Cancer, Leukemia, Lymphoma Multiple Myeloma and Plasma Cell Neoplasm, Myelodysplastic Syndromes, Neuroblastoma, Ovarian Carcinoma, Sarcoma Testicular Germ Cell Tumor	peripheral blood lymphocyte therapy	• Determine the feasibility of donor lymphocyte infusion as adoptive immunotherapy	/	Completed
Cancer Research UK	NCT01212887	Breast Cancer, Colorectal Cancer, Gastric Cancer, Lung Cancer, Ovarian Cancer, Pancreatic Cancer, Unspecified Adult Solid Tumor, Protocol Specific	MFE23 scFv-expressing autologous anti-CEA MFEz T lymphocytes, aldesleukin, cyclophosphamide and fludarabine phosphate	evaluate the feasibility assess the toxicity of this regimen in these patients. determine the dose required to give optimal survival of these cells in the circulation (recommended phase II dose).	assess whether MFE23 scFv-expressing autologous anti-CEA MFEz T lymphocytes isolated from the circulation are functional. determine the preliminary tumor response evaluate the safety	Terminated
University of Washington	NCT00228358	HER2-positive Breast Cancer, Recurrent Breast Cancer, Recurrent Non-small Cell Lung Cancer Recurrent Ovarian, Epithelial Cancer, Recurrent Ovarian Germ Cell Tumor, Stage IV Breast Cancer, Stage IV Non-small Cell Lung Cancer, Stage IV Ovarian Epithelial Cancer, Stage IV Ovarian Germ Cell Tumor	ex vivo-expanded HER2-specific T cells	• Feasibility and Safety of infusing HER2 specific T cells	Number of patients in whom the precursor frequency of antigen specific T cells is increased by 10-fold over baseline within one week after the last infusion Number of patients in whom an immune response is demonstrated if baseline immune response was below detection • HER2 specific CD4+ or CD8+ precursor frequencies • Anti-tumor effects of HER2 specific T cells • Persistence of T cell immune augmentation in vivo after adoptive transfer of HER2 specific T cells	Completed
Adaptimune	NCT01567891	Recurrent Ovarian Epithelial cancer	Citoreductive surgery followed by infusion with NYESO-1(C259) transduced autologous T cells.	To determine the safety and tolerability of autologous redirected T cell therapy	Tumor Response	Recruiting

More recently, the University Health Network of Toronto has focused its attention on patients With Platinum Resistant High Grade Serous Ovarian, Fallopian Tube, or Primary Peritoneal Cancer, starting a Phase I Study to evaluate the Feasibility and Safety of "Re-Stimulated" Autologous TILs infusion, followed by low-dose IL-2 (NCT01883297). Patients will receive an intravenous infusion of autologous TILs, previously collected from each patient, stimulated ex-vivo with autologous dendritic cells (DCs) and OKT3 (anti-CD3 antibody), and then given back to the patient. After infusion of TILs, low-dose IL-2 therapy will be given. This study, for which results are expected in 2023, has the primary objective to assess the number occurrences and severity of side effects; it also aims at measuring the clinical response to treatment and the number of patients with an immunity and no immunity to the study treatment.

In 2015 the Harlev Hospital has started a Phase I Study in which 6 patients affected by metastatic cancer will receive autologous TIL infusion after 1 week of preconditioning chemotherapy with cyclophosphamide and fludarabile. TIL infusion will be followed by IL-2 administration to support T-cell activation and proliferation. This Study aims to determine the safety of this type of therapy and results are expected for Jul 2017.

## HLA unrestricted adoptive immunotherapy

In this section, we will focus on natural and ex vivo generated immune effectors capable of exerting antitumor activity in a HLA-unrestricted way. Namely, we will focus on natural Killer (NK), lymphokine-activated Killer (LAK) and Cytokine-induced killer (CIK) cells.

### NK cells and LAK cells

Natural killer cells (NK cells) are involved in innate immunity and tumor surveillance; they also have the ability to recognize major histocompatibility complex (MHC) class I or class I-like molecules on target cells through a unique class of receptors, NK cell receptors (NKR), that can inhibit or activate NK cell function [25]. NK cells represent about 10 % of circulating lymphocytes, with a CD56 + CD3- mature phenotype and wield their activity through MHC-independent mechanisms [43]. NK cells can be divided in CD56^bright^ CD16^−^ population, which are characterized by low cytotoxicity but are able to produce [44] high amounts of cytokines, and CD56^dim^ CD16^+^ population which mediate antibody-dependent cellular cytotoxicity (ADDC) through CD16 [45].

NK cells activation is dependent upon the activation of costimulatory NK cell receptors (NKR), including NKG2D, DNAX accessory molecule-1 (DNAM-1), 2B4, NTB-A, CRACC, CD2, CD59, NKp80, CD94/NKG2C, and of the natural cytotoxicity receptors (NCR: NKp30, NKp44, and NKp46) [46]. NK cells have a well-known capacity to kill a wide variety of tumors, like sarcoma and leukemia [47–49] but in ovarian cancer disease the efficacy of NK cells to kill tumor is not clear [1]. Carlsten et al. have shown that NKs derived from healthy donor can recognize and kill in vitro ovarian carcinoma cells, isolated from peritoneal effusions, through the activation of DNAM-1 signaling with complementary contributions of NKG2D and NCR receptors [46]. On the contrary in 1984 it has been shown that NK cells derived ascitis from patients affected by EOC did not have cytotoxic potential against autologous tumors and NK cells derived from same patients showed reduced cytotoxic activity against K-562 cell line [49]. In 2005 Clarke’s group demonstrated that CA125 (that is expressed by EOC and it is used to monitor disease progression after therapeutic intervention [50]) is a potent inhibitor of NK cell-mediated cytolysis of tumor cells [44] through the downregulation of CD16 and CD94/NKG2A expression. They showed that NK cells incubated with CA125 for 72 h exhibited a 50–70 % decrease in the lysis of K562 targets respect to control [44]: The same group in 2007 published a work which demonstrated that NK cells derived from ascites were enriched in CD56^bright^ CD16^−^ subset compared to NK cells derived from autologous peripheral blood (32 versus 10 %) [51]. Lymphokine Activated Killer cells (LAK) cells are a heterogeneous mixture of ex vivo expanded and activated T, NK and NKT cells which display major histocompatibility complex (MHC)-non-restricted cytotoxicity that do not rely on HLA-mediated recognition of tumor targets. Thesenatural effectors carry out their antitumoral activities without identify and recognize the presence of specific TAA expressed on the cells surface; HLA-unrestricted immunotherapy approach don’t involve TCR engineering or CAR type therapies . Phillips JH et al. showed the activity of LAK cells against several cancer cell lines, such myelogenous leukemia line (K562) and colon cancer cell lines [52]. In the eighties, Rosenberg’s group first reported the use of LAK cells to treat 25 patients with advanced cancer: patients received both autologous LAK cells with high doses of interleukin-2. They observed objective regression in 11 of the 25 cases with pulmonary or hepatic metastases from melanoma, colon cancer, or renal-cell cancer and complete tumor regression was observed in primary unresectable lung adenocarcinoma. Unfortunately the administration of high doses of IL2 was the cause of a strong toxicity limiting the use of LAK cell therapy [53].

Shuen-Kue group demonstrated that non-cytotoxic and sublethal pretreatment of ovarian cancer cell line (Skov-3 cells that are not sensitive to NK cells) with paclitaxel enhance LAK cell-mediated killing [54].

### CIK cells

Cytokine-induced killer cells (CIKs), are heterogenous ex vivo expanded T lymphocytes [55], characterized by the presence of two main subsets: the first, with a CD3 + CD56+ phenotype, mainly responsible for the anti-tumor activity of CIKs and the second (CD3 + CD56-) more similar to conventional T lymphocytes [56]. The antitumor activity of CIKs is MHC-unrestricted and mostly mediated by the interaction of CIKs' membrane receptor NKG2D with MHC class I polypeptide-related sequence MICA, MICB or members of the unique long 16-binding protein (ULBP) family (ULBP1, 2 and 3) on tumor cells [57–59]. The expression of these proteins is potentially induced by pathological stimuli and it is reported to be associated with several tumor histotypes [56]. Initial clinical applications demonstrated clinical activity of CIKs in many solid cancers, such as non–small-cell lung cancer, hepatocellular cancer, renal cell cancer and gastric cancer, confirming a very favorable safety profile [60, 61].

Retrospective studies have shown that NKG2D ligands are expressed on the surface of ovarian cancer cells. Li and colleagues analyzed 82 EOC patients, finding that MICA/B and ULBPs are expressed in 97.6 and 82.9 % of samples respectively and that high expression of ULBPS is an indicator of poor prognosis [62]. Similar results were also reported by McGilvray and colleagues who showed that the expression of l RAET1 and ULBP2 NKG2D ligands correlates with a worse prognosis [63].

Preclinical studies support the antitumor activity of CIK cells against ovarian cancer both in vitro and in vivo.

Gritzapis and collegues have shown the possibility to expand CIK cells from peripheral blood of patients with ovarian cancer in the presence of appropriate cytokines such as IL2, IFNγ and OCT3 [64]. CIK cells demonstrated in vitro cytotoxic activity against autologous targets with a perforin-mediated action [64]. In other preclinical models, CIKs were able to kill 45 % of SK-OV-3 human ovarian cancer cells and were able to significantly inhibit 73 % of SK-OV-3 tumor growth in nude mice xenografts [65].

An interesting preclinical approach, based on antibody-redirection of CIK cells, was reported by Negrin’s group. The authors explored CIK-mediated killing of primary ovarian carcinoma with and without bispecific antibodies against cancer antigen-125 (BSAbxCA125 with affinity to both CD3 and CA125) and Her2 (BSAbxHer2, with affinity to both CD3 and Her2). Addition of bispecific antibodies significantly enhanced the mean percentage of tumor specific killing in vitro models. These results were confirmed in vivo. Adoptive infusion of CIK cells plus bispecific antibodies (BSAbxCA125 or BSAbxHer2) resulted in significant reduction of tumor burden and prolongation of survival compared to controls [66].

#### Clinical activity of NK, LAK and CIK cells agains ovarian cancer

Geller and colleagues have recently studied in vivo expansion and efficacy of adoptively transferred allogeneic NKs in 14 ovarian and 6 breast cancer patients after a lymphodepletion regimen [67]. The preparative regimen consisted in high-dose cyclophosphamide and fludarabine, in seven cases followed by a 200 cGy total body irradiation (TBI). Lymphodepletion was previously shown to increase innate immunity through higher homeostatic cytokines exposure (like IL-7 and IL- 15) and reduction of the T regulatory and myeloid-derived suppressor cells number [68, 69]. All patients had failed four or more prior therapies for metastatic disease. NK cell product was obtained from haplo-identical related donors, incubated overnight in 1000 U/mL interleukin (IL)-2 prior to infusion. A median cell dose of 2.15 × 10^7^ NK cells/kg (range 8.33 × 10^6^- 3.94 × 10^7^) was infused two days after the last dose of chemotherapy and, in the same day, patients began the subcutaneous IL-2 administration (three times weekly for a total of six planned doses). Most patients reported expected grade 1 and 2 toxicities but there were also 10 unexpected severe adverse events, including a grade 5 toxicity. The death, was probably related to a tumor lysis syndrome associated with NKs therapy. Moreover, two cases of passenger lymphocyte syndrome and an autoimmune hemolytic anemia were reported. With the addition of TBI, Geller and colleagues observed improvement of hematopoietic recovery [67].

As clinical activity against EOC following NKs infusion, 4 patients had a partial response (PR), 8 a stable disease (SD) and 1 a progressive disease (PD); the median time to progression (TTP) was 2 months. As IL-15 level is considered a good candidate to drive NKs expansion, based on previous reports [70], Geller and colleagues showed that IL-15 levels were increased in serum after lymphodepletion regimen in comparison with baseline. However, it began to fall after two weeks (day +14) [67]. Similarly, at the end of IL-2 therapy (day +14 after NKs infusion), the donor NKs were soon replaced by recipient T regulatory (T-reg) cells. No improvement in rates of NKs expansion was reported in patients treated with additional TBI. Several hypotheses for the limited NKs proliferation were proposed: T cell immune rejection; suppression by myeloid derived suppressor cells; suppression by T-reg cells. Geller and colleagues suggested that the use of exogenous IL-2 to increase in vivo NKs expansion could promote also a host Tregs raising. In order to overcome IL-2 side effects and to avoid Tregs expansion, they support the use of IL-15 as a more NK-selective cytokine [67].

In 1990 Stewart et al. evaluated the safety and activity of LAKs in 10 patients with chemo-resistant ovarian cancers. Patients were previously treated with 6 intraperitoneal infusion of IL-2. Mononuclear cells were collected by leukaphereses and LAKs were reinfused in peritoneum with IL-2 followed by 3 additional doses of IL-2. The dose-limiting toxicity was the accumulation of ascites and the consequent abdominal pain; other adverse reactions were fever, nausea and vomiting, diarrhea, anemia (red blood cells transfusion was necessary in all cases) and performance status decrease. The reported clinical response was poor and nine patients had a disease progression; only one patient had a partial response, followed by disease progression after 3 months [71].

The role of maintenance therapy with CIK cells was recently tested by Liu and colleagues. In a phase II study the authors investigated the feasibility and the efficacy of this therapy by measuring progression free survival (PFS) and overall survival (OS). Ninety two patients with stage IIB- IV EOC were enrolled; all of them underwent cytoreductive surgery followed by 6–8 courses of carboplatin and paclitaxel chemotherapy. One month after the last course, 46 patients received monthly infusion of 11.82 ± 1.61X 10^9^ autologous CIK cells, while the other 46 patients received no further treatment. A significant increase in median PFS was observed in patients treated with CIKs as maintenance therapy (37.7 vs 22.2 months, *p* = 0.004). This advantage was confirmed in all the subgroup analyzed. OS did not reach statistical significance, except in stages IIB-IIIB subgroup analysis, even if there was a trend in favor of the CIKs-treated arm. Liu et al. speculate the possible influence by the second line therapy, the small number of patients and the inappropriate follow-up time. No severe toxicities related to the CIKs infusion were reported; the most common adverse reaction was grades 1 and 2 pyrexia [55].

## Conclusions

Adoptive cell-therapy has been shown to be an active treatment for different kinds of cancers, such as melanoma and other solid and hematological malignancies [31, 60, 61, 66, 72, 73]. The demonstration that the presence of inflammatory infiltrate correlates with a better prognosis for patients with EOC, suggests that it may be a relevant tool also in the treatment of EOC [6].

Results of several preclinical studies indicate that both HLA unrestricted immune effectors and HLA restricted T-lymphocytes have a cytotoxic activity against EOC cells in vitro [46, 64].

However, clinical research is still at an early stage and only few evidences of efficacy of adoptive immunotherapy in EOC have been reported, in particular regarding the addition of a maintenance therapy with CIK or TIL to front line standard treatments. This additional therapy seems to be able to improve clinical outcome prolonging PFS and OS in patients with a newly diagnosed EOC [42, 55]. On the contrary, the few published clinical trials with NK in other clinical settings such as multi-resistant EOC, were not able to demonstrate any activity of adoptive immunotherapy.

An Important issue that may significantly influence the outcome of clinical ACT is the employment of preparative lymphodepleting regimens. The scope of such treatments is to eliminate potentially immune-suppressive elements and create an appropriate “immunologic space” for the incoming immune effectors, reducing their competition for sustaining cytokines. Currently, there is no agreement on what may be the optimal lymphodepleting regimen. Combinations of Cyclofosfamide, Fludarabine and low dose total body irradiaton (TBI) seem to provide the best results and are therefore explored in clinical trials [23, 28].

The definition of the most suitables T cell subtypes for ACT is current object of intense research efforts; differentiation state of CD8+ T cells is inversely related to their capacity to proliferate and persist. These findings may be clinically relevant, and younger T cells are statistically positively correlated with clinical effectiveness in ACT trials [23, 28].

One of the possible limits to the clinical employment of adoptive immunotherapy is represented by the complexity of the procedures involved in this kind of treatments. Cell therapy must be individualized, because the therapeutic agent is represented by patient's own cells which have to be collected, expanded and finally re-infused, with every step performed in GMP (Good manufacturing practice) validated facilities according to rigorous and stringent regulations. Personalization of adoptive cell-therapy, however, can represent an advantage: each tumour has different biological and molecular features and immunotherapies based on the use of autologous cells, have potential of high specificity, not achievable with chemotherapy.

Adoptive immunotherapy seems to be generally well-tolerated and toxicities reported are especially related to use of cytokines (such as IL-2) to promote cellular expansion.

Other important potential toxicities associated with ACT may be due to undesired antigen-recognition in healthy organs or to massive cytokine storm even if such events appear more likely to occur with genetically redirected lymphocytes [74].

Furthermore, the systemic administration of IL-2 might induce an undesired in vivo expansion of T regulatory cells (Treg) that may counteract the beneficial effect of ACT [23].

In melanoma, the presence of TILs was shown to be functionally linked to clinical benefit obtained with checkpoint inhibitors such as antibodies blocking CTLA-4 and PD1 molecules [75–77]. Preclinical evidence on the importance of PD-1 expression in Tumor-infiltrating NY-ESO-1-specific CD8+ T cells is also available [78]. In relapsed resistant/refractory ovarian cancer anti PD-1/PD-L1 antibodies have shown promising activity with favourable safety profile [79, 80].

On these bases, adoptive immunotherapy may potentially synergize with checkpoint inhibitors treatments. This future perspective may be even more applicable with genetically redirected T lymphocytes as supported by encouraging preclinical evidence [81, 82].

NK seems to be related to more severe toxicities, the only clinical trial published, investigating NK efficacy in patients with multi-resistant EOC reported a death for tumor lysis syndrome, two cases of passenger lymphocyte syndrome, an autoimmune haemolytic anaemia and no improvement of clinical outcome [67]. Although the prognostic role of NK cells infiltration is still controversial, with published article that suggest their negative prognostic role [83].

In conclusion, application of adoptive cell therapy against EOC appears as a promising perspective, not yet sufficiently supported by convincing clinical data. It seem reasonable that adoptive cell therapy may provide the best benefit in settings of low tumor burden, minimal residual disease, or maintenance therapy. These concepts should be incorporated and integrated in the multidisciplinary therapeutic strategy of ovarian cancer [84].

The complexity and costs required to explore clinical applications of these approaches remain open issues that may be faced if supported by further and stronger preclinical evidences.

Further studies are therefore needed to better define the patterns involved in the immune response to EOC and the escape mechanisms allowing neoplastic cells survival and proliferation, in order to develop strategies to make adoptive immunotherapy clinically effective.

### Ethics approval and consent to participate

Not applicable.

### Consent for publication

Yes.

### Availability of data and materials

Yes.
